# Supplementary material for: Sub-surface thermal measurement in additive manufacturing via machine learning-enabled high-resolution fiber optic sensing
Source: Nat Commun. 2024 Aug 31;15:7568. doi: 10.1038/s41467-024-51235-7 (PMC11365934; doi:10.1038/s41467-024-51235-7)
Supplement: Supplementary file 1 — Supplementary Information [file 41467_2024_51235_MOESM1_ESM.pdf]

Supplementary Information

Sub-surface thermal measurement in additive manufacturing via machine learning-enabled high-resolution fiber optic sensing

Rongxuan Wang[1], Ruixuan Wang[2], Chaoran Dou[3], Shuo Yang[4], Raghav Gnanasambandam[3], Anbo Wang[2], Zhenyu (James) Kong[3]*

Department of Industrial and Systems Engineering, Auburn University[1]

Bradley Department of Electrical and Computer Engineering, Virginia Tech[2]

Grado Department of Industrial and Systems Engineering, Virginia Tech[3]

Department of Biomedical Engineering, Washington University in Saint Louis[4]

*Corresponding author (zkong@vt.edu)

I. C-FBG Training Dataset (dimension: 800 points × 2000 frames)

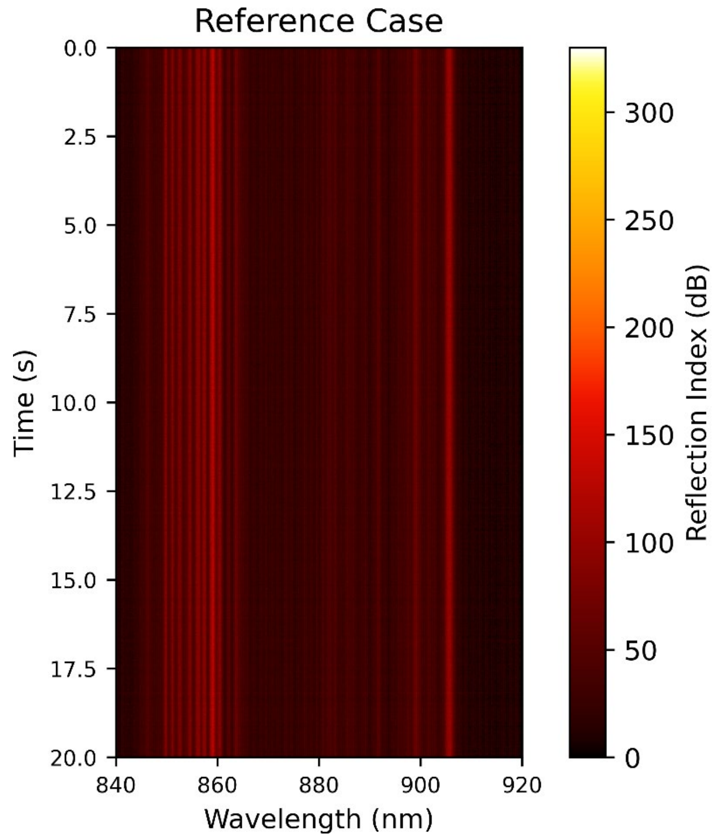

Supplementary Figure 1. C-FBG Training dataset reference case visualization. Source data are provided as a Source Data file.

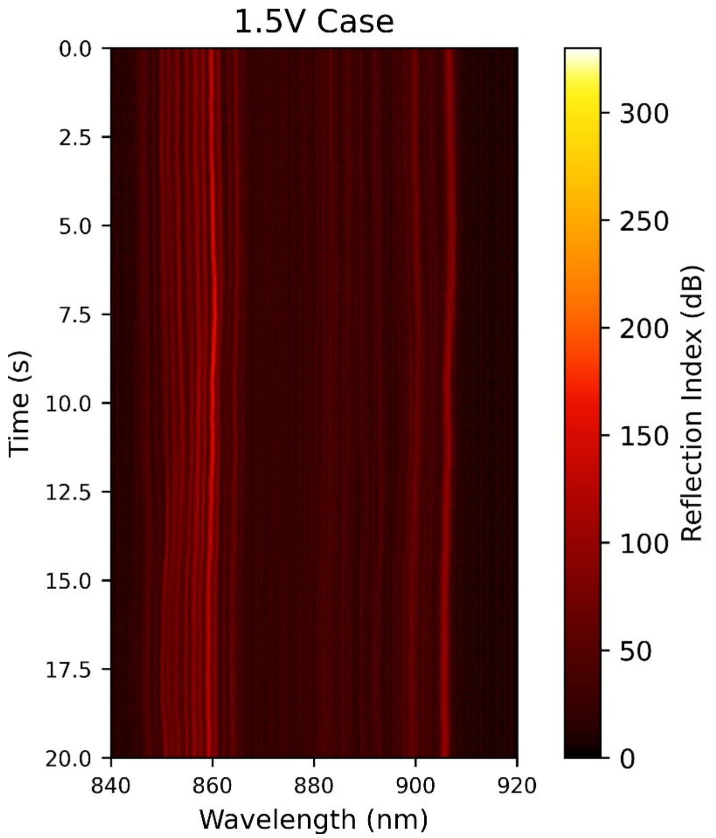

Supplementary Figure 2: C-FBG Training dataset 1.5V case visualization. Source data are provided as a Source Data file.

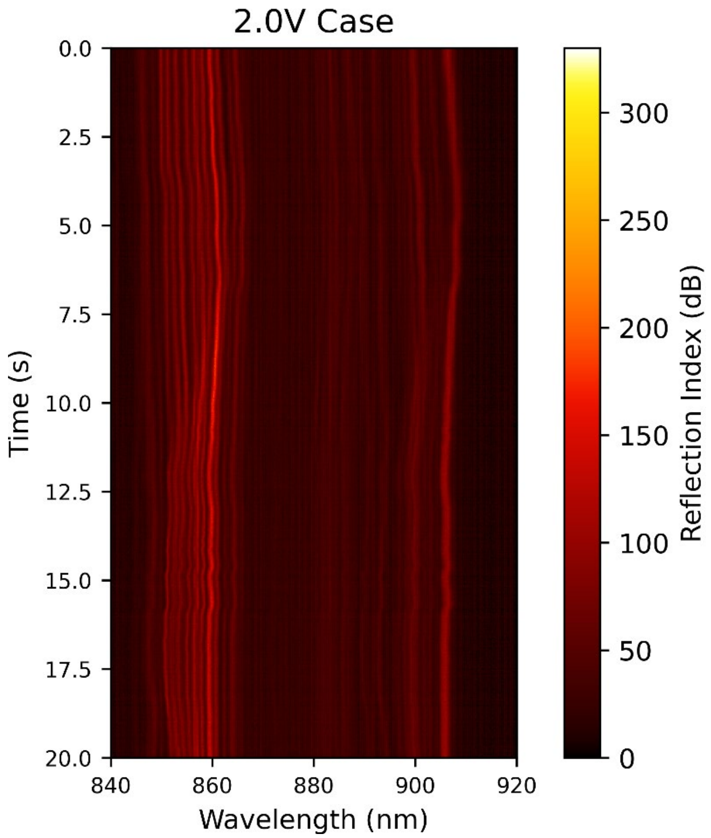

Supplementary Figure 3: C-FBG Training dataset 2.0V case visualization. Source data are provided as a Source Data file.

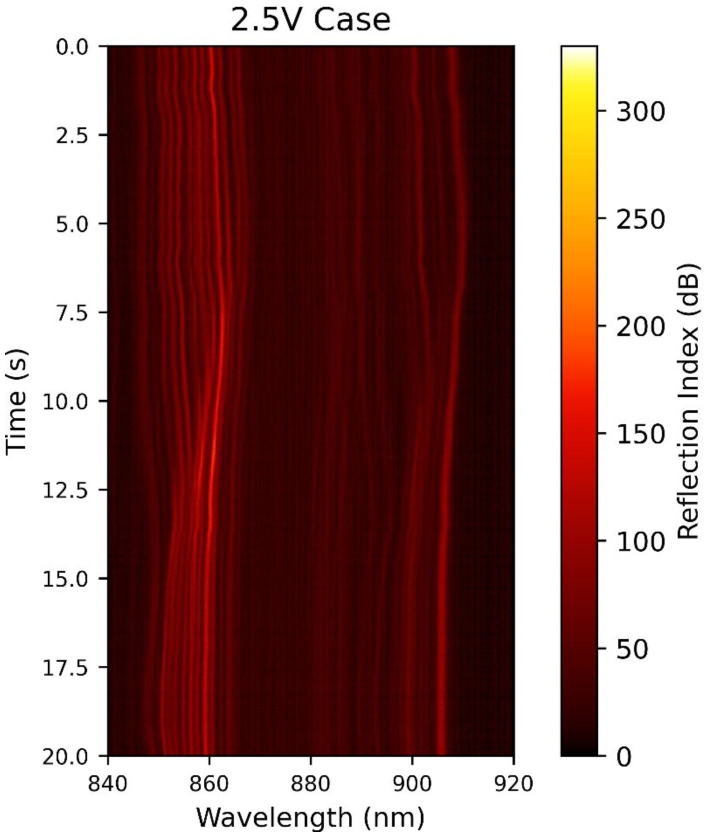

Supplementary Figure 4: C-FBG Training dataset 2.5V case visualization. Source data are provided as a Source Data file.

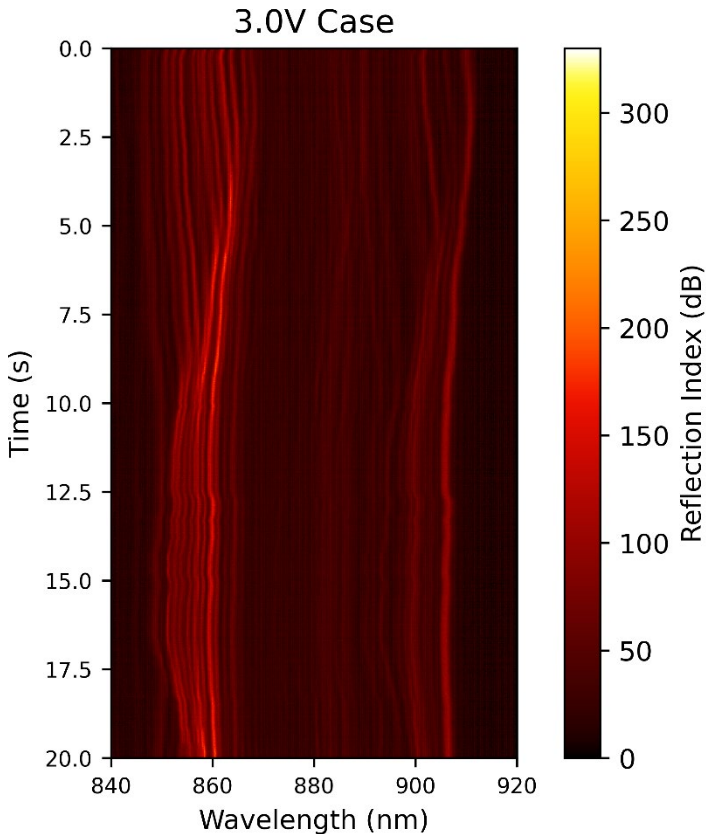

Supplementary Figure 5: C-FBG Training dataset 3.0V case visualization. Source data are provided as a Source Data file.

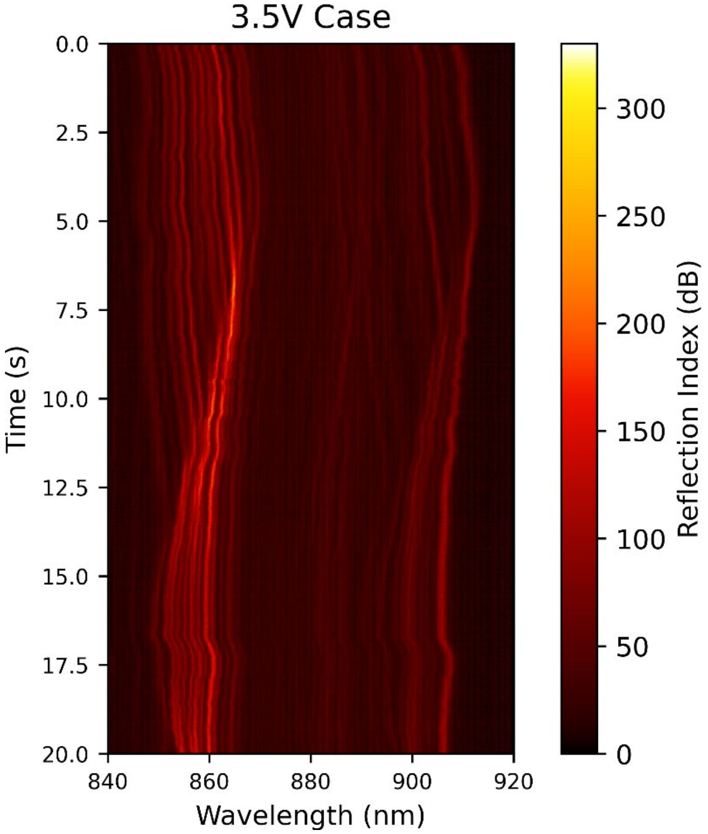

Supplementary Figure 6. C-FBG Training dataset 3.5V case visualization. Source data are provided as a Source Data file.

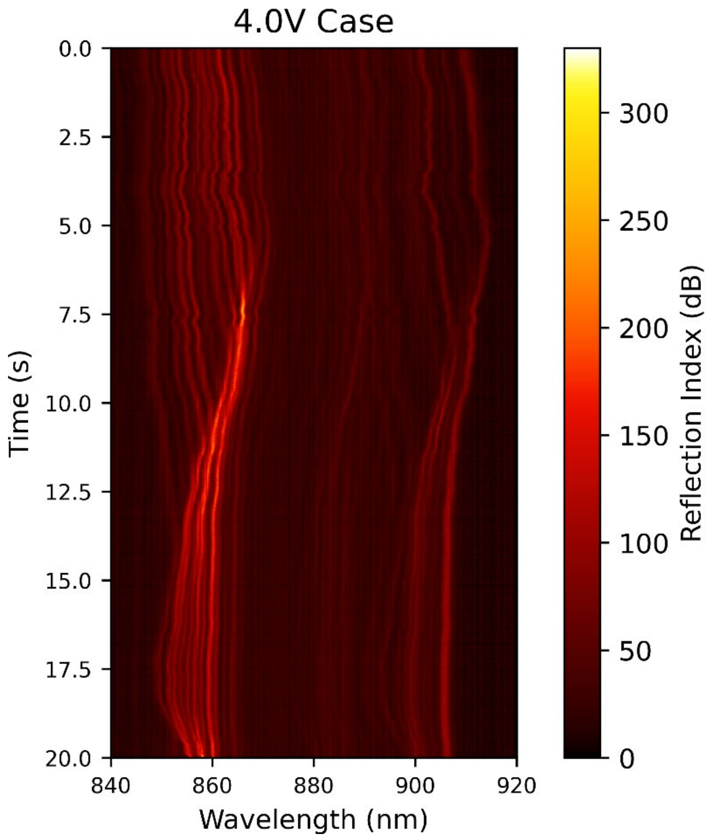

Supplementary Figure 7. C-FBG Training dataset 4.0V case visualization. Source data are provided as a Source Data file.

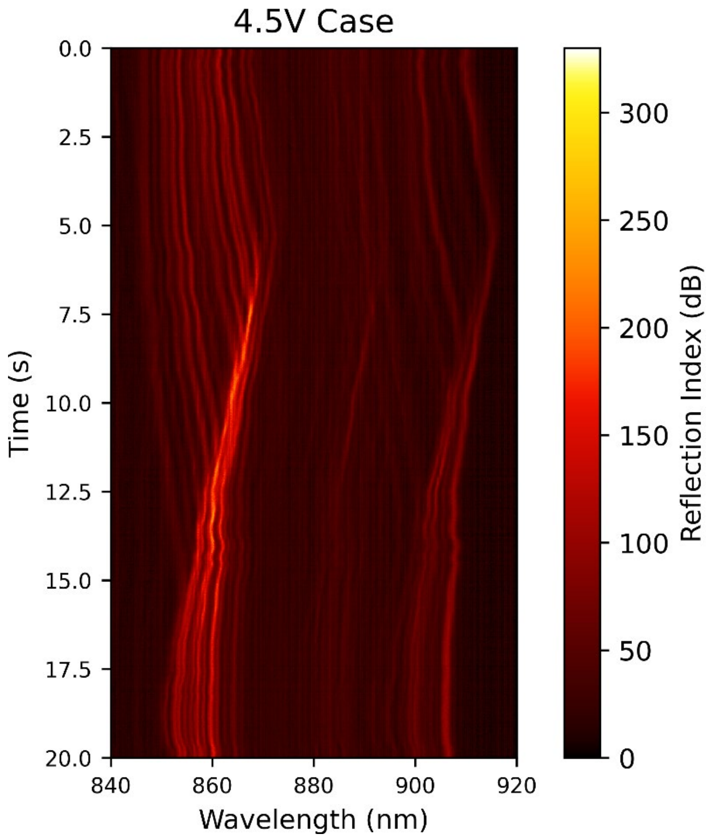

Supplementary Figure 8. C-FBG Training dataset 4.5V case visualization. Source data are provided as a Source Data file.

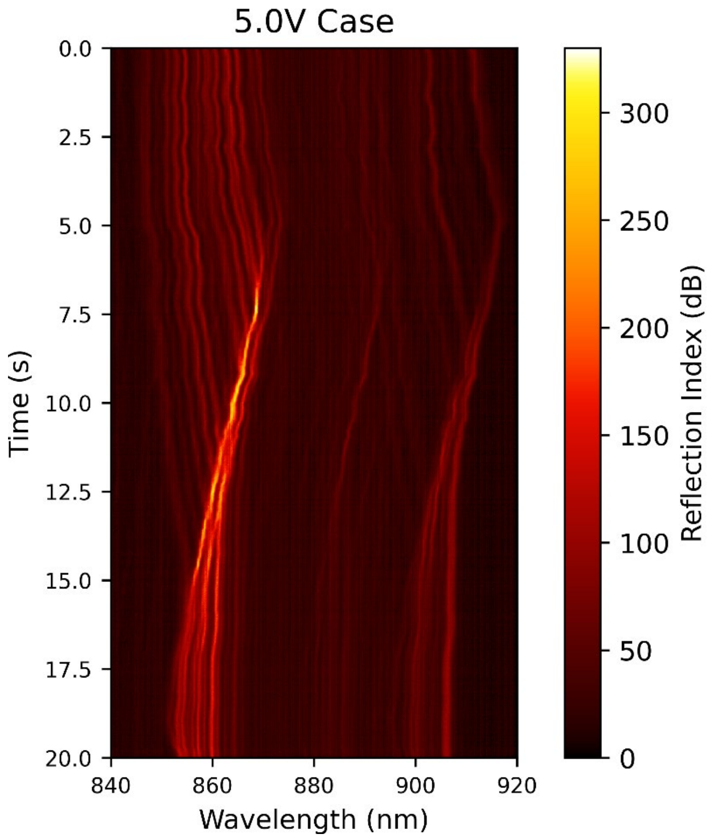

Supplementary Figure 9. C-FBG Training dataset 5.0V case visualization. Source data are provided as a Source Data file.

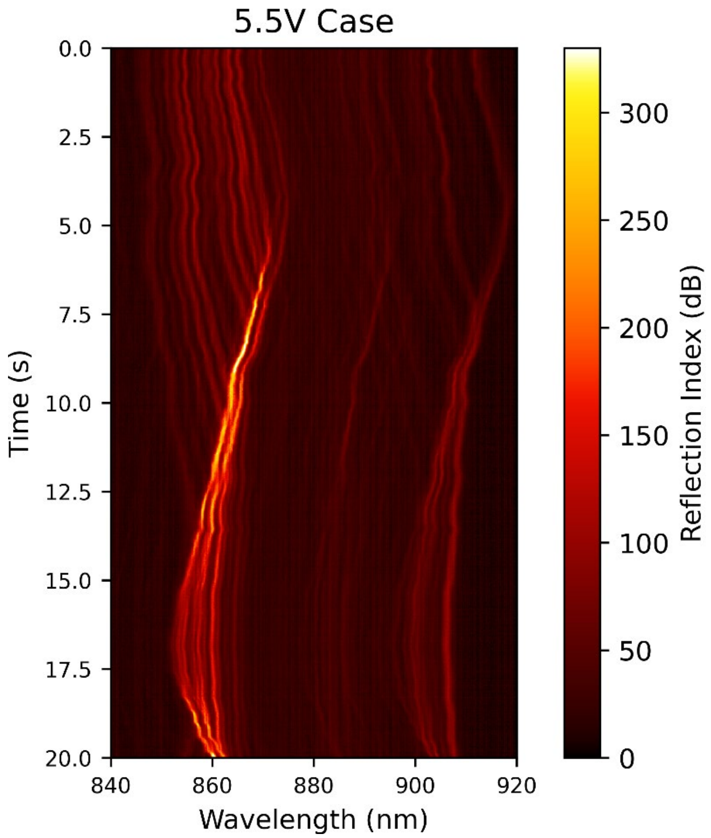

Supplementary Figure 10. C-FBG Training dataset 5.5V case visualization. Source data are provided as a Source Data file.

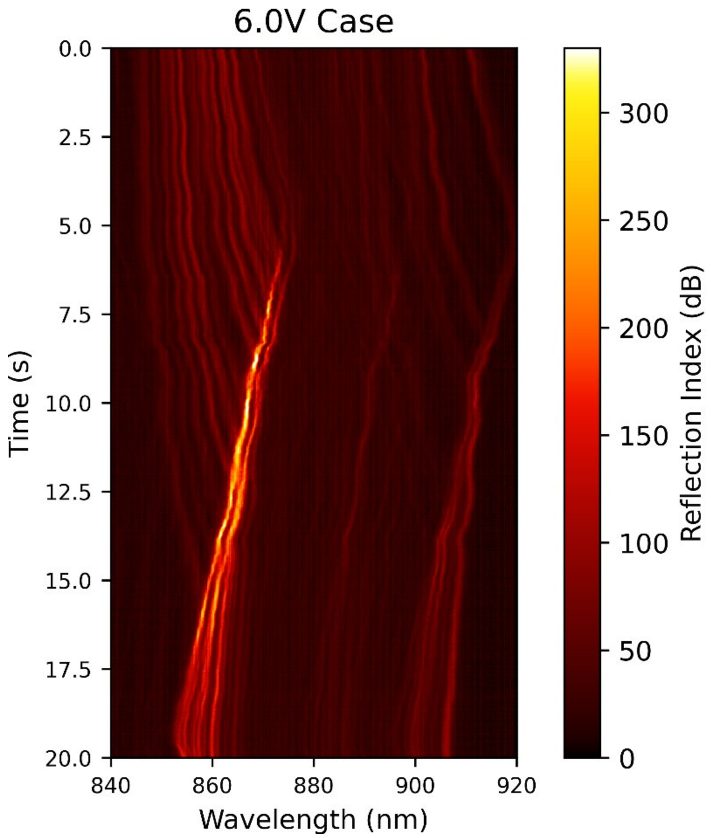

Supplementary Figure 11. C-FBG Training dataset 6.0V case visualization. Source data are provided as a Source Data file.

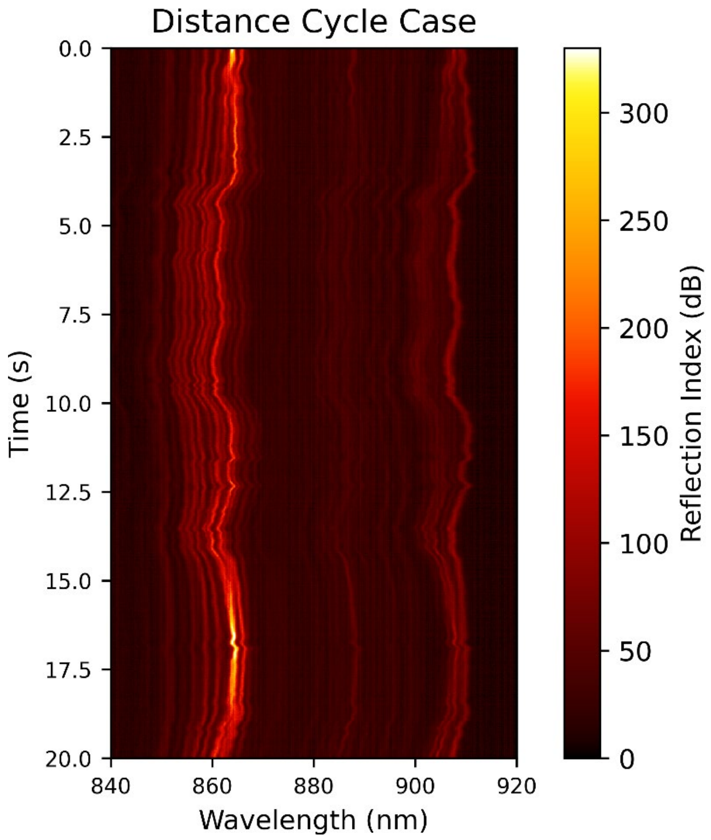

Supplementary Figure 12. C-FBG Training dataset distance cycle case visualization. Source data are provided as a Source Data file.

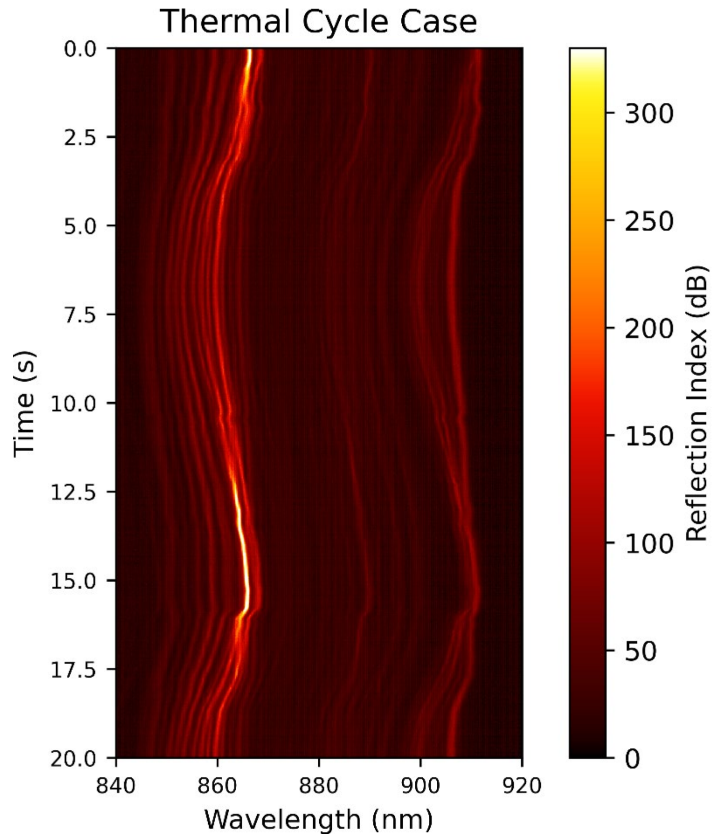

Supplementary Figure 13. C-FBG Training dataset thermal cycle case visualization. Source data are provided as a Source Data file.

II. Thermal Profile Training Dataset (dimension: 480 points × 2000 frames)

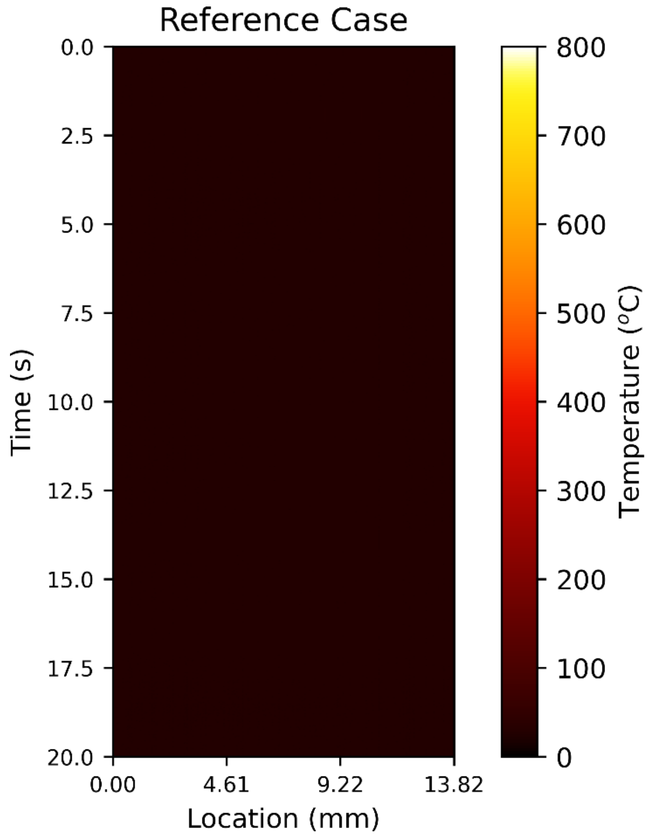

Supplementary Figure 14. Thermal profile training dataset reference case visualization. Source data are provided as a Source Data file.

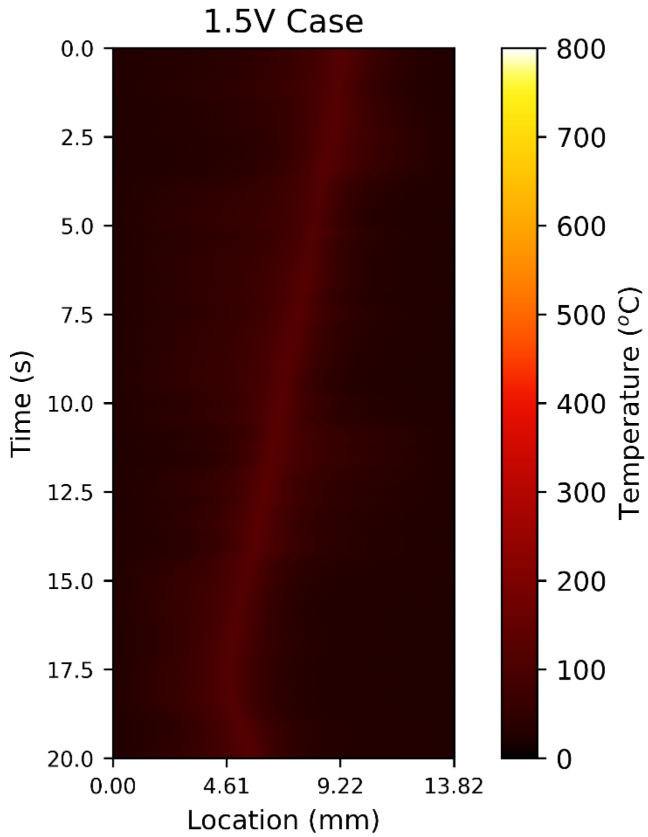

Supplementary Figure 15. Thermal profile training dataset 1.5V case visualization. Source data are provided as a Source Data file.

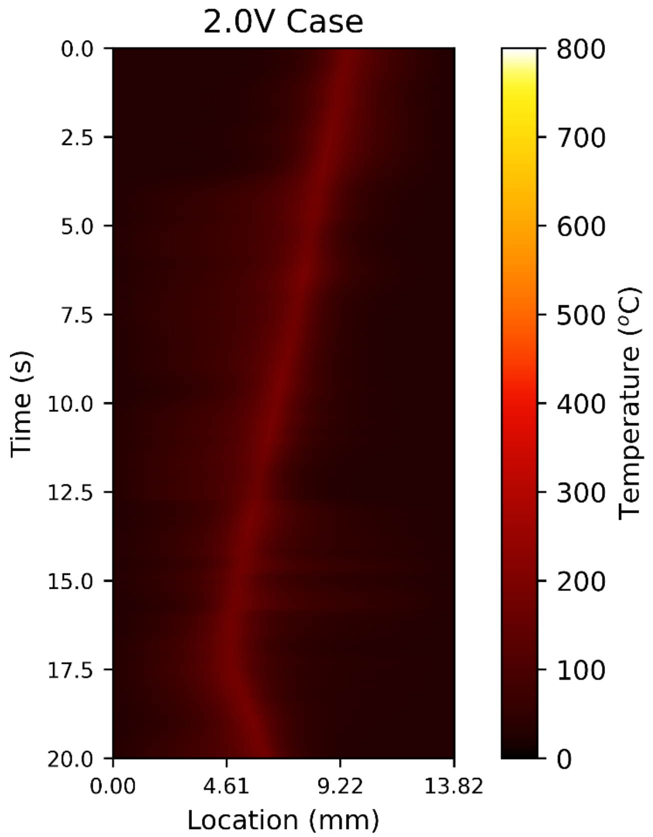

Supplementary Figure 16. Thermal profile training dataset 2.0V case visualization. Source data are provided as a Source Data file.

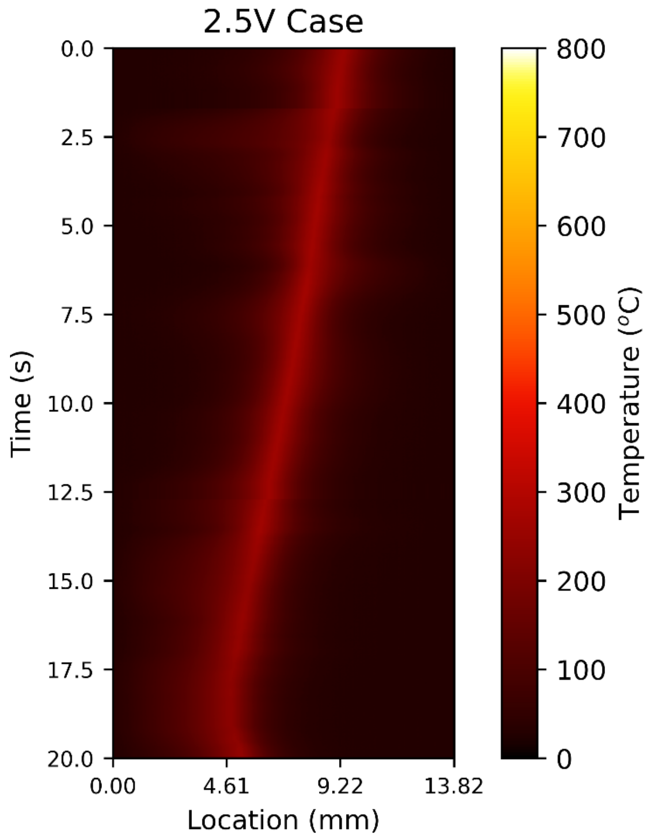

Supplementary Figure 17. Thermal profile training dataset 2.5V case visualization. Source data are provided as a Source Data file.

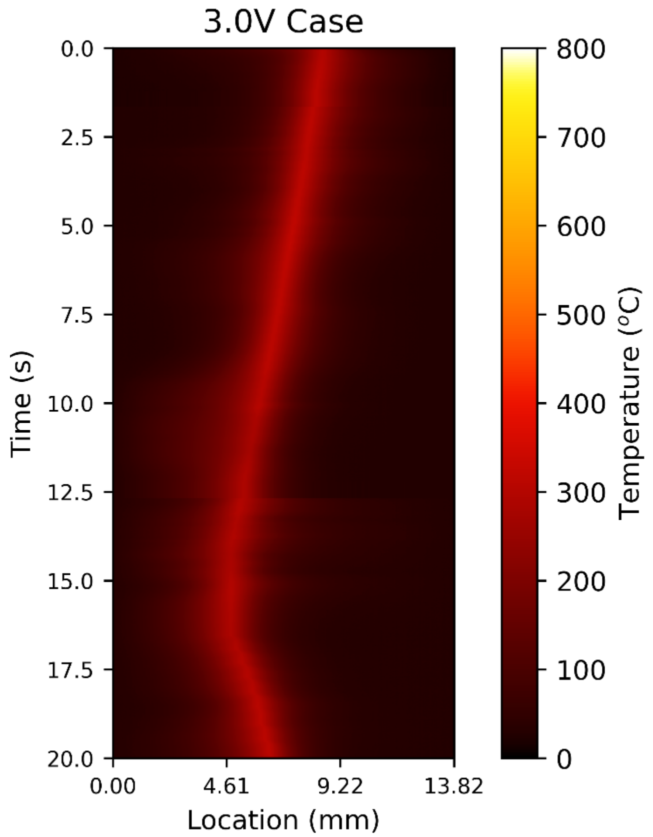

Supplementary Figure 18. Thermal profile training dataset 3.0V case visualization. Source data are provided as a Source Data file.

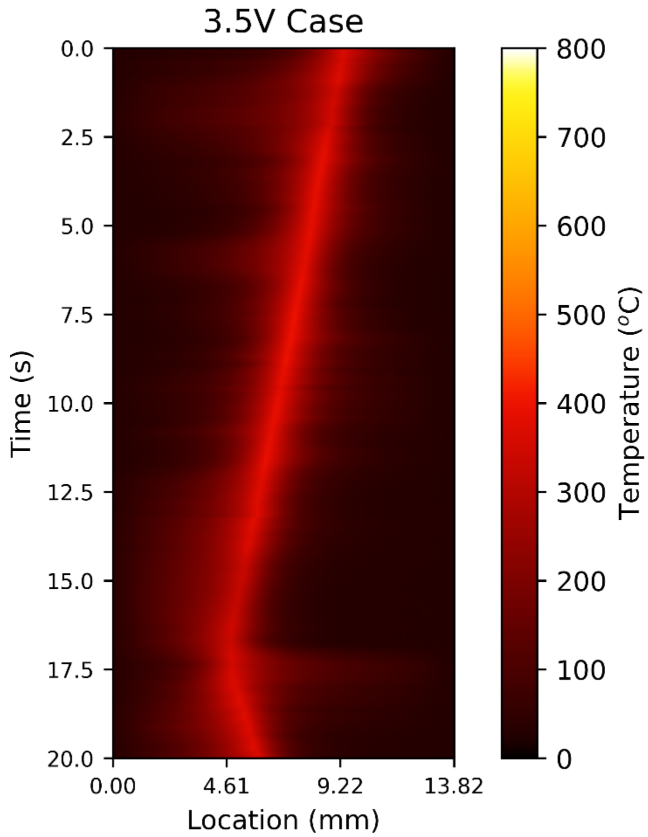

Supplementary Figure 19. Thermal profile training dataset 3.5V case visualization. Source data are provided as a Source Data file.

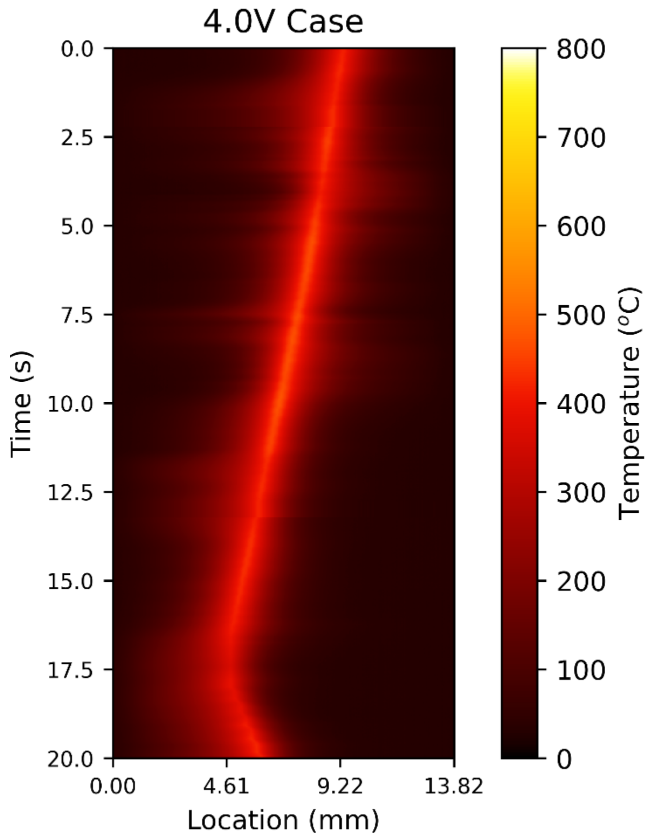

Supplementary Figure 20. Thermal profile training dataset 4.0V case visualization. Source data are provided as a Source Data file.

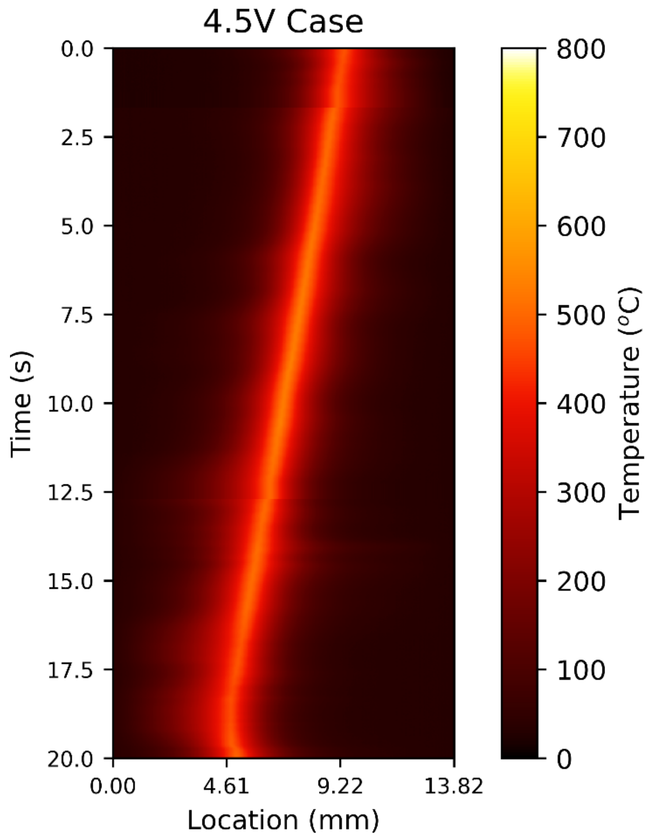

Supplementary Figure 21. Thermal profile training dataset 4.5V case visualization. Source data are provided as a Source Data file.

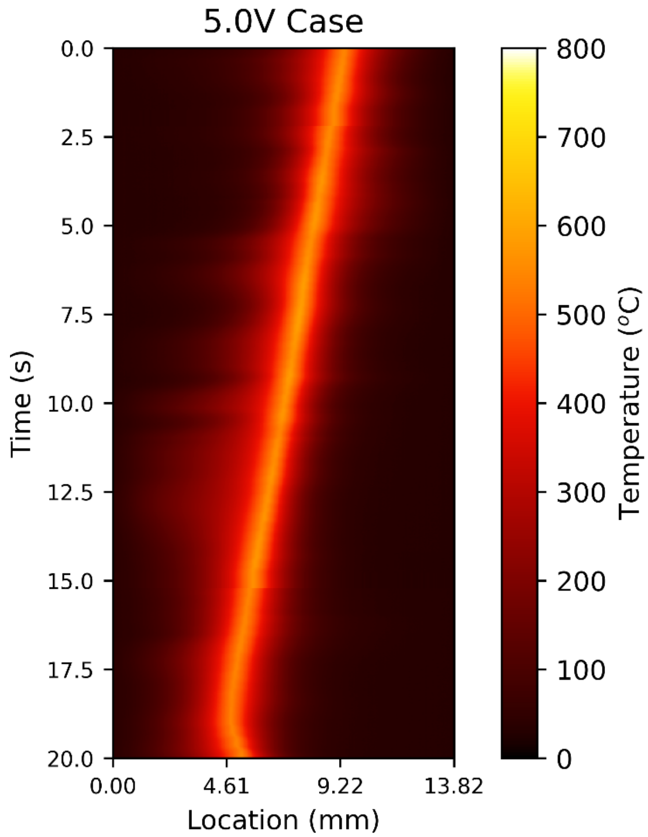

Supplementary Figure 22. Thermal profile training dataset 5.0V case visualization. Source data are provided as a Source Data file.

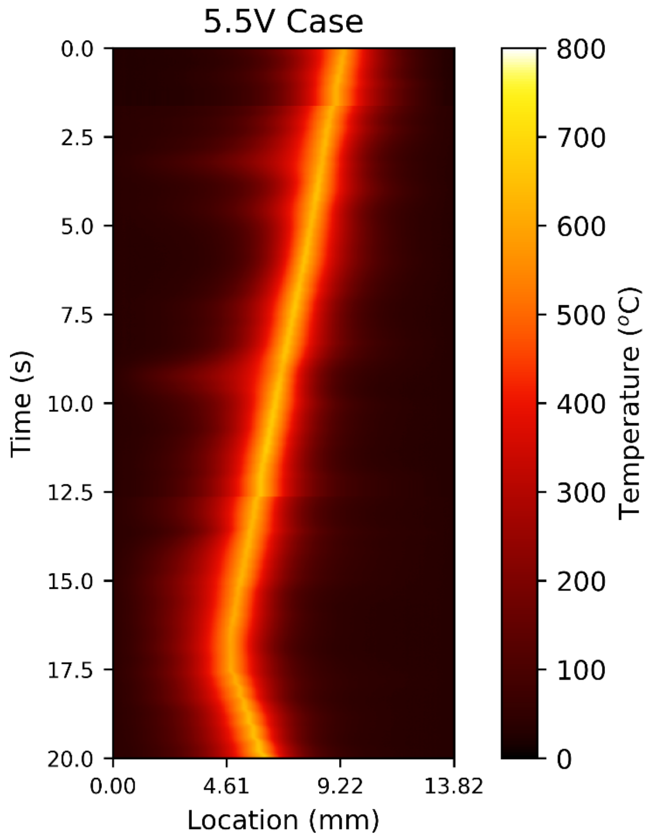

Supplementary Figure 23. Thermal profile training dataset 5.5V case visualization. Source data are provided as a Source Data file.

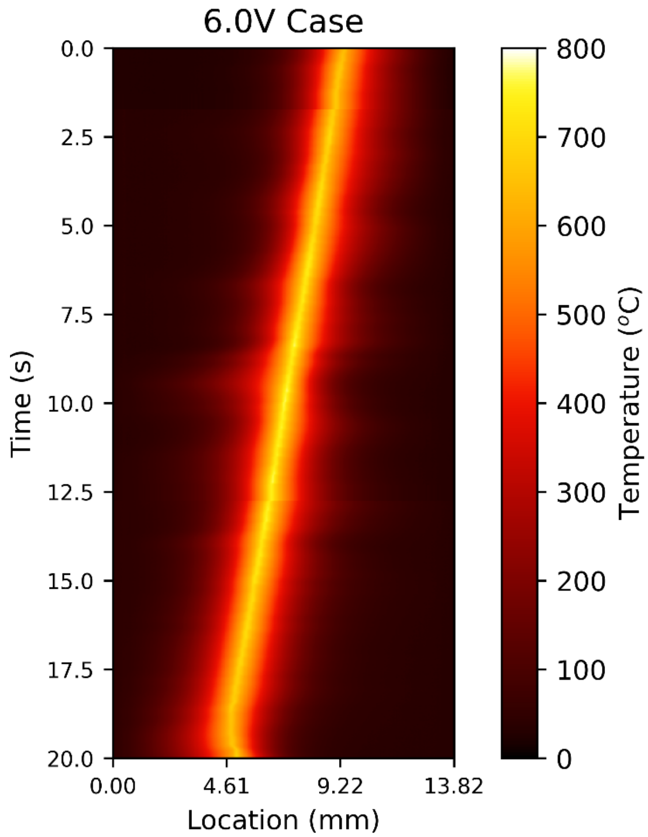

Supplementary Figure 24. Thermal profile training dataset 6.0V case visualization. Source data are provided as a Source Data file.

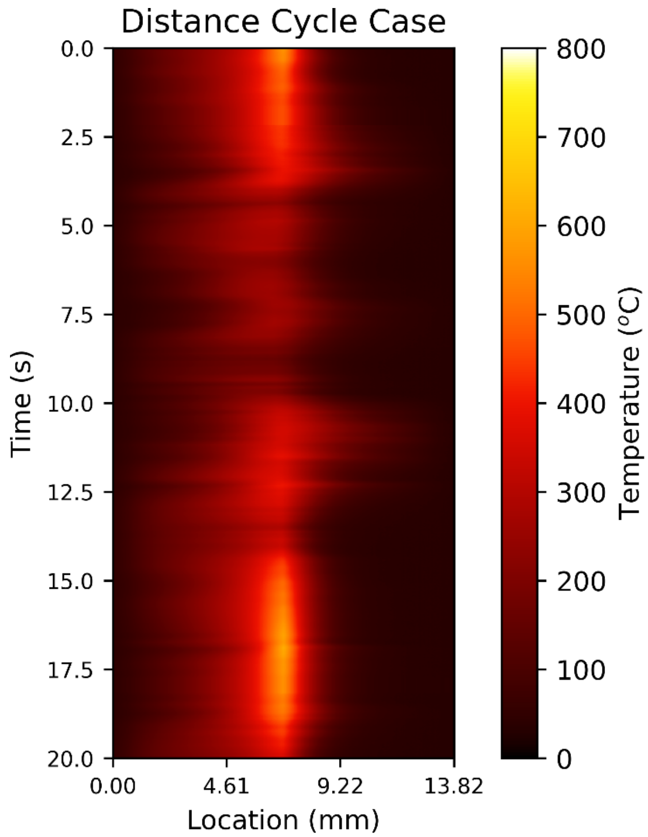

Supplementary Figure 25. Thermal profile training dataset distance cycle case visualization. Source data are provided as a Source Data file.

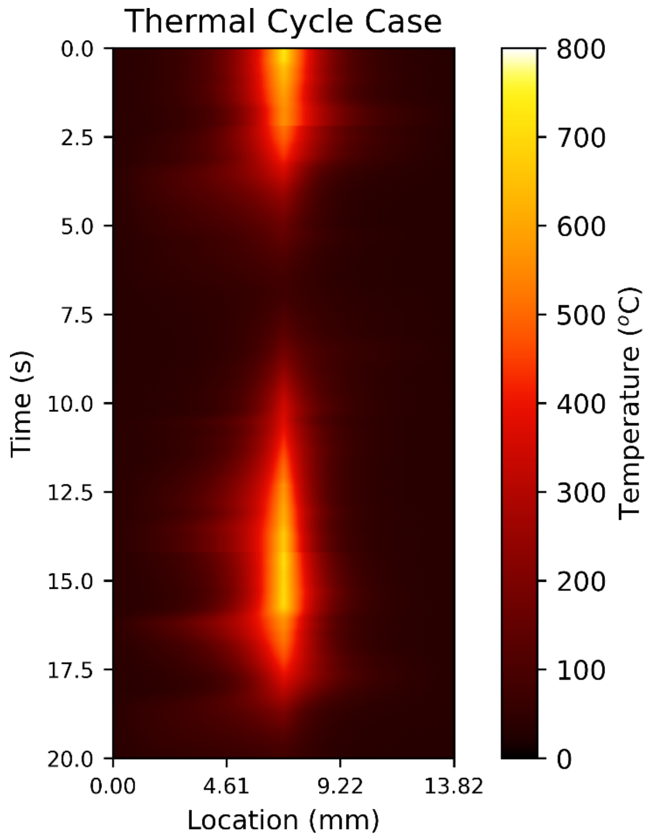

Supplementary Figure 26. Thermal profile training dataset thermal cycle case visualization. Source data are provided as a Source Data file.
